# Beyond the role of the reproductive caste of chicatana ants (*Atta mexicana*) as an alternative protein source: nutritional, food safety, and bioactivity analysis

**DOI:** 10.3389/fnut.2026.1868210

**Published:** 2026-09-30

**Authors:** Vicente A. Mirón-Mérida, Ivan Luzardo-Ocampo, Mariana Martínez-Avila, Víctor H. Urrutia-Baca, Arlette Santacruz, Eduardo Enrique Gallareta-Flores, Marilena Antunes-Ricardo, Cristina Chuck-Hernandez, Esther Pérez-Carrillo

**Affiliations:** 1 Tecnologico de Monterrey, School of Engineering and Sciences, Monterrey, Mexico; 2 Tecnologico de Monterrey, Institute for Obesity Research, Monterrey, Mexico

**Keywords:** anti-inflammatory activity, antioxidant activity, chicatana ant (*Atta mexicana*), entomophagy, fatty acids, insect flour, insects

## Abstract

**Introduction:**

Mexican leaf-cutter ants (*Atta mexicana*), also known as chicatana ants, have been consumed in Mexico since pre-Columbian times as a seasonal food commodity and delicacy. Most studies on this edible insect have focused on its nutritional value as a protein source. Because of its proximate composition, this insect can also be considered a source of other important macro- and micronutrients. However, due to its geographic location and collection practices, food safety risks may be present.

**Methods:**

In this study, chicatana ants were collected in Huatusco, Veracruz, Mexico, and analyzed to determine their nutritional value, the presence of heavy metals and pathogens, and potential compositional bioactivity related to lipids and phenolic compounds.

**Results and Discussion:**

Proximate analysis showed the importance of chicatana ants not only as a protein source (38.16%) with an adequate protein digestibility (79.06%) but also as a source of fat (36.54%), mainly composed of saturated and unsaturated fatty acids such as oleic (57.02%), palmitic (21.94%), stearic (14.05%), and linoleic acid (6.98%). Additionally, chicatana ants provide chitin (16.53%), fiber (12.85%), and minerals (1.91%), including microelements such as calcium (532.14 mg/kg), iron (93.92 mg/kg), and zinc (161.09 mg/kg). Selected concentrations (500 and 750 μg/ml) displayed cytoprotective effects under pro-oxidant conditions in Caco-2 cells and anti-inflammatory effects in polarized THP-1 macrophages, as indicated by significant nitrite reduction. Although freeze-drying did not reveal microbial contamination, lead levels exceeded the maximum permitted levels for some European Union-approved insect species (0.1 mg/kg), reinforcing the need to explore additional food safety concerns for chicatana ants and other edible insects.

**Conclusion:**

The results highlight the nutritional and biological potential of *A*. *mexicana* lipids and phenolic compounds for manufacturing functional foods with these health benefits. However, the study also identified food safety concerns. Further research into specific biological mechanisms is necessary to confirm these effects and elucidate bioaccumulation mechanisms for contaminants and toxic compounds.

## 1 Introduction

Entomophagy is part of Mexico's history, as pre-Hispanic cultures attributed symbolic and medicinal value to edible insects, a tradition that has endured, especially in rural areas and among ethnic communities, in which approximately 549 species are consumed (1). Mexican leaf-cutter ants (*Atta mexicana*), also known as “Chicatana,” are consumed in both rural and urban areas. People perceive this species differently: some consider it an agricultural pest, but many communities value it as a food commodity (2, 3). Winged females of the reproductive caste are annually harvested during the rainy season between May and June, when the winged males and females leave their anthill to reproduce, also known as nuptial flight. People eat female ants that will become queens because they are larger and more flavorful, while males often emit a strong odor that some people associate with urine and are often called “mionas.” During the rainy season, people widely consume chicatana ants, especially in Oaxaca, Chiapas, Veracruz, Puebla, Morelos, Tlaxcala, and Guerrero. Although no harvesting or processing guidelines exist, residents in these areas usually collect ants from the soil or pavement, wash them in water, and roast them on a flat pan (comal). They usually remove the wings during or after roasting, and depending on the practice, some people might remove the abdomen or wings or even roast them while still alive. Consumption patterns vary by region. Whole chicatana ants are eaten in Chiapas and Oaxaca, whereas only the headless bodies or abdomens are considered edible in Veracruz and Morelos (4). These ants are consumed either as complete snacks or incorporated into culinary dishes, including sauces, dips, creams, pizza, tamales, moles, and Mexican snacks such as “Antojitos mexicanos” (1).

Based on initial studies, chicatana ants are regarded as a protein source (5). Other studies suggest exploring this edible insect as a source of fiber, soluble carbohydrates, and fatty acids that support body maintenance and may also provide health benefits (6). For instance, the protein concentrate from *A*. *mexicana* has shown antioxidant and hypoglycemic activities (4). Other studies have shown the antimicrobial and neurological activities of its peptides (7). Chicatana ant is also a source of lipids, among which saturated fatty acids are major components (8), and its fatty acid profile remains unaffected after roasting (9).

Despite their potential bioactivity, people mainly consume chicatana ants for their sensory properties (flavor) and tradition (10), and some studies report that knowledge of their consumption has been lost over time (11). Because current studies mainly focus on the bioactivity of the protein fraction of chicatana ants, a research gap remains regarding the benefits of other nutritious fractions, such as lipids, fiber, and antioxidant compounds. Besides, studies on the risks associated with wild harvesting remain scarce. In this study, the nutritional value of the reproductive caste, precisely the adult females of the Chicatana ant, was assessed by analyzing and characterizing their nutritional components and assessing the bioactivity of their phenolic compounds and lipids. Furthermore, the presence of microorganisms and heavy metals was assessed for the first time in *Atta mexicana*, as food safety parameters. This work aims to address knowledge gaps on edible chicatana ants, particularly regarding the bioactivity of lipid and phenolic content and the importance of food safety analysis in edible insects.

## 2 Materials and methods

### 2.1 Ant collection

Chicatana ants were harvested from the cloud forest of Huatusco, Veracruz, Mexico (latitude: 19.14666667 °N, longitude: −96.95 °W, altitude: 1,186 m.a.s.l.), on June 27th, 2023, by a local collector. This humid mountainous region is also known for its coffee production. Collection was carried out according to the collector's traditional practices, which involved handpicking the ants from the forest floor. In June 2023, Huatusco's average monthly temperature and precipitation were 20.5 °C and 114.5 mm, respectively, according to records from station number 30342 of the National Water Commission (CONAGUA, 2023). After collection, samples were stored in the collector's home freezer at −18 °C and transported to the laboratory in an ice-filled box, where they were washed with tap and distilled water. The wings were manually removed, and the body was lyophilized for 4 days (moisture content: 6.54 ± 0.24 %). All analyses were performed on ant flours after grinding the whole body (Krups grinder, Millville, NJ, USA).

### 2.2 Proximate analysis

The proximate composition of the samples, namely moisture, ash, and protein contents, was determined following official AOAC methods (AOAC 925.10, 923.03, and 984.13, respectively) (12). For the protein content, a nitrogen-to-protein conversion factor of 6.25 was used. The total fat content was quantified using the standardized Goldfish method (13), and the total dietary fiber (TDF) content was determined gravimetrically using a commercial enzymatic dietary fiber kit (TDF100A, Sigma-Aldrich, St. Louis, MO, US), following the manufacturer's instructions.

### 2.3 *In vitro* protein digestibility analysis

Alkaline protein extraction was conducted on a whole ant flour dispersion (1:10 w:v, flour: distilled water) at pH 9. After incubation at 72 rpm for 1 h at 52 °C, samples were centrifuged at 10,000 rpm for 10 min at 23 °C. The pH of the supernatant was adjusted to pH 4.5 with HCl (15% v/v), and the supernatant was centrifuged as previously described. The pellet was frozen at −80 °C and freeze-dried for 4 days. The protein content was determined as indicated in section 2.2 (14).

The *in vitro* protein digestibility (IVPD) was determined in a 50-ml solution (6.25 mg of protein/ml) at pH 8. Samples were incubated with 5 ml of a solution containing trypsin (1.6 mg/ml, 1,000–2,000 BAEE/mg solid, T4799; Sigma-Aldrich, St. Louis, MO, USA), type II α-chymotrypsin from bovine pancreas (3.1 mg/ml, ≥40 U/mg protein, C4129, Sigma-Aldrich), and Type XIV protease from *Streptomyces griseus* (1.3 mg/ml, ≥3.5 U/mg protein, P5147, Sigma-Aldrich) at 37 °C for 10 min (15). The initial and final pH values were recorded, and IVPD and relative protein digestibility were calculated using the following equations:


$$\begin{array}{l} IVPD\;\left(\%\right)=210.46-18.1\;x\;\left(pH\;at\;minute\;10\right) \end{array}$$

$$\begin{array}{l} Relative\;Protein\;Digestibility=\frac{pH\;sample}{pH\;casein}x\;100 \end{array}$$

### 2.4 Quantification of the chitin content

The chitin content was gravimetrically quantified by incubating ant flour (500 mg) with HCl (1 M, 10 ml) at 85 °C for 50 min. The samples were then centrifuged (3,000 × g, 10 min), and the precipitates were washed with distilled water (10 ml) and centrifuged twice. Washed pellets were incubated with NaOH (1 M, 10 ml) at 90 °C for 35 min. The mixture was vacuum-filtered through a Whatman #1 filter (11 μm pore size), washed 3 × (distilled water), and dried overnight (105 °C). The residue was quantified as chitin (16).

### 2.5 Fatty acid analysis by GC-MS and enrichment metabolomics

The fatty acid profile of the oil extracted from the ants was obtained by gas chromatography–mass spectrometry (GC–MS) following the method described by Martínez-Ávila et al. (17). Briefly, the lipid fraction of the ant flour was extracted with hexane in an orbital shaker (MaxQ 5000, Thermo Scientific, Waltham, MA, USA) at 170 rpm for 3 h at room temperature (22 ± 1 °C) using an ant flour: hexane ratio of 1:10 (w:v). Oil extracts were derivatized to their corresponding fatty acid methyl esters (FAMEs) following a standardized transesterification procedure (AOAC, method 996.06).

The resulting FAMEs were separated on a PerkinElmer Clarus gas chromatograph (PerkinElmer, Shelton, CT, USA) coupled to a mass spectrometer, using a TG-5 MS capillary column (30 m × 0.25 mm, 0.25 μm) and helium (flow rate: 1 ml/min) for a total run time of 32.33 min. MS was performed with an electrospray ionization source at 70 eV. The quadrupole temperature was set at 150 °C, and the interface temperature at 200 °C. Mass spectra were acquired over a range of m/z = 30–550 UMA using TurboMass 6.1.1.2019 software. Fatty acids were identified by comparing retention times with the standards and confirmed in the NIST mass spectral library. Bioinformatics analysis of the obtained GC-MS composition of the ant fat obtained by the Goldfish and Soxhlet methods was conducted using MetaboAnalyst 6.0. We used an untargeted metabolomics approach with metabolite set enrichment analysis, considering the top 25 metabolites enriched in metabolic pathways, with pathways classified by enrichment ratios and *p-*values.

### 2.6 Micro-element analysis by ICP-MS

A MARS 5 microwave (CEM, Matthews, NC, USA) was used to digest 0.5 g of insect flour in 10 ml of concentrated nitric acid for 20 min at 180 °C. The resulting colorless samples were cooled to room temperature (22 ± 1 °C), and the volume was adjusted to 25 ml with Milli-Q water. The samples were then filtered with Whatman No. 41 filter paper.

For elemental quantification, inductively coupled plasma mass spectrometry (ICP-MS; Agilent Technologies, St. Louis, MO, USA, 7800) was performed with a 50 s purge and stabilization time, a 3-point peak pattern, and 100 cycles in triplicate. Calibration curves were obtained for calcium (Ca), manganese (Mn), iron (Fe), copper (Cu), zinc (Zn), arsenic (As), selenium (Se), cadmium (Cd), mercury (Hg), and lead (Pb), with a liquid multi-element standard product containing 24 inorganic elements (AccuStands Co., New Haven, CT, USA). Concentrations were reported in mg/Kg and calculated by multiplying each interpolated concentration (ng/ml) by the total volume (25 ml) and dividing by the sample weight on a dry basis (0.47 g). The limits of detection (LOD) and quantification (LOQ) ranged from 0.001–5 ng/ml and 0.005–250 ng/ml, respectively. Each specific LOD and LOQ value is indicated in Supplementary Table S1, and the calibration curves are shown in Supplementary Figures S1–S10 (18, 19). LC-MS grade water was used as a blank, and 10 consecutive measurements were used to determine the LOD and LOQ according to the following equations:


$$\begin{array}{l} LOD=\left(3.3\;X\;SD\right)/k \end{array}$$

$$\begin{array}{l} LOQ=\left(10\;X\;SD\right)/k \end{array}$$

where SD is the blank's standard deviation (*n* = 10), and k is the slope of each calibration curve.

### 2.7 Microbiological assays

Prepared serial dilutions of 1 g of flour in 9 ml of sterile peptone water. Triplicate dilutions were plated on Petri dishes containing either nutrient or Eosin Methylene Blue (EMB) agar and incubated under aerobic conditions at 37 °C for 48 h to quantify aerobic mesophilic microorganisms and total coliforms, respectively. Samples were placed on potato dextrose agar (PDA) and incubated at 23 °C for 7 days to quantify molds and yeasts. To validate the results, we plated three dilutions (10^−2^, 10^−4^, 10^−6^) in triplicate. Plates with counts ranging from 30 to 300 CFU ml^−1^ were considered for the assays. For reproducibility, each triplicate was performed at three different times.

### 2.8 Quantification of the total phenolic compounds and antioxidant capacity

Free total phenolic compounds were extracted from the whole and defatted flours, which were prepared following the procedure describe by Abdel-Aal and Hucl (20) with slight modifications. Briefly, the whole and defatted flours (0.5 g) were mixed with 10 ml of 1% HCl-methanol, and blends were left under constant stirring (250 rpm) in the dark at 22 ± 1 °C for 18 h. Samples were then centrifuged at 4 °C (3,000 *g* for 10 min) and filtered (0.45 μm acrodiscs).

To determine the ants' fat content, an ethanolic extract was generated by ultrasonication as reported by Zhang et al. (21). For this, the fat (1.0 g) was mixed with 10 ml of cold 80% v:v ethanol, and the solution was sonicated 10 times in 1-min intervals, maintaining the solution at 4 °C using an ice bath. All samples were protected from light.

Total phenolic compounds were quantified by the Folin–Ciocalteu method using a gallic acid standard curve, and results were expressed as mg equivalents of gallic acid per g of sample (GAE/g) (22). For antioxidant capacity, two methods were used: the 2,2-diphenyl-1-picrylhydrazyl (DPPH) and 2,2′-azino-bis (3-ethylbenzothiazoline-6-sulfonic acid; ABTS) radical-scavenging assays. The DPPH method was performed as described by Brand-Williams et al. (23), and the ABTS method was performed as indicated by Nenadis et al. (24), both using a Trolox standard curve (0–800 μm). Results were reported as μm Trolox equivalents per g of sample. The antiradical activity (ARA), expressed as a percentage, was calculated using the following formula:


$$\begin{array}{l} ARA\;\left(\%\right)=\left(1-\frac{Abs_{sample}}{Abs_{control}}\right)x\;100\% \end{array}$$

where Abs corresponds to the absorbance of either the sample or the control (DPPH + Methanol alone).

### 2.9 Cell culture analyses

#### 2.9.1 Treatment preparation for cell culture

Before cell treatment, the samples were pre-treated to facilitate their solubilization in cell culture media. Extracts of the whole and defatted flours were prepared as indicated in section 2.8. However, the obtained supernatants were concentrated in a vacuum concentrator. Briefly, 1 ml of supernatant was placed in a microcentrifuge tube and vacuum-dried in a vacuum centrifuge (Vacufuge Plus, Eppendorf, Hamburg, Germany) at 20 hPa for 1 h at 45 °C. Each tube was reconstituted with 1 ml of 2% Fetal bovine serum (FBS) in Dulbecco's Modified Eagle's Medium (DMEM, vortexed until completely dissolved, and filtered through a sterile 0.22-μm filter. Serial dilutions were prepared to obtain solutions at 62.5, 125, 500, and 750 μg/ml. These concentrations were based on previous reports of non-cytotoxic concentrations for other insect flours, as no reports on *A*. *mexicana* were found for treating human colon adenocarcinoma Caco-2 cells (25, 26). All treatments were used at 37 °C to avoid cell stress.

Considering that the macromolecules, including the fat, of the ants are potential heavy metal bioaccumulators (27), the ants' fat was also used in the cytotoxicity assays. Following extract preparation as reported in section 2.8, the fat was solubilized in the cell culture media as reported by Zhang et al. (21). The previously obtained cold solution (100 μl) was added to cold 2% FBS-DMEM to dissolve it, and the ethanol concentration was maintained below 0.5% to avoid cell death. To avoid entrapment of lipid micelles, the solubilized ants' fat solution in sterile DMEM was not filtered; all procedures were conducted under sterile conditions using sterile materials in a level 2 biosafety cabinet. Fat treatments were used only with Caco-2 cells to assess cytotoxicity, whereas whole and defatted flour-based extracts were used in both cell lines (Caco-2 and polarized human monocytes, THP-1).

#### 2.9.2 Metabolic activity under basal and pro-oxidant conditions in Caco-2 cells

Human epithelial-like colorectal adenocarcinoma Caco-2 [Caco2] (ATCC HTB-37) cells were purchased from the American Type Culture Collection (ATCC, Manassas, VA, USA). The cells were cultured using Dulbecco's Modified Eagle's Medium (DMEM; Gibco, Thermo Fisher Scientific, Waltham, MA, USA) with glucose (25 mm), NaHCO_3_ (3.7 g/L), 10% FBS (Gibco), and 1% antibiotics (penicillin and streptomycin, Gibco). Cells were maintained in a humidified 5% CO_2_ environment at 37 °C until they reached 80% confluence.

For basal metabolic activity, cells were cultured in 96-well plates for 24 h (1 × 10^4^ cells/well) to promote attachment. The growth medium was then discarded, and 100 μl of each treatment (whole and defatted flour-based extracts and fat at 62.5, 125, 250, 500, and 750 μg/ml, prepared in 2% FBS) was added. After 24 h of treatment, the medium was replaced with a 3-(4,5-dimethylthiazol-2-yl)-2,5-diphenyltetrazolium bromide (MTT) solution (0.5 mg/ml, prepared using only DMEM, without antibiotics or FBS; Invitrogen, ThermoFisher Scientific) in sterile, antibiotic-free 2% FBS-DMEM, and the cells were incubated for 1.5 h under typical cell growth conditions. The medium was then removed, and dimethyl sulfoxide was added (100 μl/well) to solubilize the formazan crystals. The plate was incubated at 22 ± 1 °C for 5 min and read at 570 nm in a Varioskan microplate reader (Thermo Fisher Scientific). The negative control consisted of cells grown in 2% DMEM with antibiotics, and the positive control consisted of cells treated with 50 mm H_2_O_2_ dissolved in 2% FBS-DMEM. The metabolic activity of the cells, expressed as a percentage of the untreated cells, was calculated as follows:


$$\begin{array}{l} Metabolic\;activity\;\left(\%\right)=\left[\frac{Abs_{sample}-Abs_{negative\;control}}{Abs_{negative\;control}}\right]\times 100\% \end{array}$$

where Abs refers to the absorbance read using the spectrophotometer.

Representative images of the cells treated at the selected concentrations were obtained using a standard inverted optical microscope (IMD-300D, Optika Microscopes, Ponteranica, Bergamo, Italy) at 10X and 40X. The metabolic activity was assessed under pro-oxidant conditions (cytoprotection) as reported by Uribe-Velázquez et al. (28). For this, in a set of independent experiments, as those conducted for cytotoxicity, the medium was replaced by a pro-oxidant solution (100 μl) containing 10 mm H_2_O_2_ dissolved in sterile 2% FBS-DMEM 30 min before using MTT.

#### 2.9.3 Impact of ant flour and fat on metabolic activity and nitrite production in differentiated THP-1 monocytes

The anti-inflammatory activity was tested in human THP-1 monocytes (ATCC TIB-202) obtained from ATCC (Manassas, VA, USA). The cells were cultured using Roswell Park Memorial Institute (RPMI) medium supplemented with 10% FBS, 1% antibiotics (100 × ), 10 mm sterile 4-(2-hydroxyethyl)-1-piperazineethanesulfonic acid (HEPES; Gibco), 1% sodium pyruvate (100 × ), and 0.05 mm 2-mercaptoethanol (Gibco). The cells were maintained under the same conditions as Caco-2 cells.

Once the cells were confluent, trypsin (0.25% trypsin/0.53 mm EDTA) was used to treat and detach the cells, and they were plated in 24-well plates to differentiate them, as previously reported (29). Briefly, cells (8 × 10^4^ cells/well) were allowed to rest for 24 h under basal conditions, and 150 nM phorbol-12-myristate 13-acetate (PMA, Gibco) was added to each well and incubated for 48 h to induce differentiation into M1 macrophages. The medium was replaced with the treatments prepared in section 2.9.2 using the whole and defatted flour-based extracts. Since the cytotoxicity of the fat at all tested fat concentrations was ≥80% after the metabolic activity of Caco-2 cells was determined, no treatments using fat were applied to THP-1 macrophages.

Once the cytotoxicity of the whole and defatted flour-based extracts was ruled out, the anti-inflammatory assay was performed to evaluate the extracts' ability to reduce nitrite production. Briefly, differentiated THP-1 macrophages were treated with extracts (62.5, 125, 500, and 750 μg/ml) prepared in sterile 2% RPMI, mixed with lipopolysaccharides (LPS) from *Escherichia coli* O111:B4 (Sigma-Aldrich) (29). After 24 h of treatment, 100 μl of the medium from each well was transferred to a 96-well plate, and 100 μl of prepared Griess reagent (1% sulfanilamide in 5% phosphoric acid and 0.1% N-1-naphthyl-ethylenediamine dihydrochloride in aqueous solution) was pipetted into each well. Plates were covered to protect them from light and incubated at room temperature (22 ± 1 °C) for 15 min. The total nitrite content was calculated from the samples' absorbances at 540 nm using a standard NaNO_2_ curve (30). For the positive control, THP-1 macrophages treated only with 2% RPMI and LPS were used, and the negative control was untreated THP-1 cells. The half-inhibitory concentration of nitrite production (IC_50_) was obtained with an equation adapted for biological reactions, following a non-linear regression, as provided by GraphPad Prism v. 10.6.1 (Dotmatics, Boston, MA, USA). Nitrite production was reported in micrograms of nanomolar equivalents of NO_2_/ml.

### 2.10 Statistical analysis

Results are reported as the mean ± standard deviation from two independent experiments, each performed in triplicate. Normality was confirmed with a Shapiro–Wilk test, the samples' residual distribution, and a test for equal variances (Bartlett or Levene's test for parametric or non-parametric data, respectively). When data were normally distributed, we conducted analysis of variance (ANOVA), with Student's/Tukey–Kramer *post-hoc* tests. For non-parametric data, a Wilcoxon/Kruskal–Wallis's test was performed, followed by a *post-hoc* Dunn's test. Differences were considered at *p*<*0*.*05*, and all analyses were performed in JMP v. 19.1.0 (SAS Institute, Cary, NC, USA).

## 3 Results and discussion

### 3.1 Proximate and microbiological analysis

Table 1 shows the proximate composition of chicatana ant flour. The calculated values were within the reported ranges for chicatana ants from different regions and collection years. In addition to being an important protein source (38.16% d.b.), chicatana ants should also be considered a source of lipids because of their similar fat content (36.54% d.b.), which is often overlooked when assessing the nutritional potential of chicatana ants. In addition to the aldehydes associated with its nutty, woody, and fatty odor (31), its fat content is closely related to its specific sensory attributes (9). A standard nitrogen-to-protein conversion factor (Kp) of 6.25 was utilized in the determination of crude protein. Although this Kp value can overestimate protein content, the complexity of different insect matrices means researchers should determine specific Kp and Ka values for each insect species (32, 33). This represents an existing gap for *Atta mexicana*, as different nitrogen-to-protein conversion factors have been calculated for more commercial species (4.93–5.76), yet all the reports for chicatana ants have utilized a 6.25 factor. This work reported two fiber types: crude fiber and TDF. The crude fiber content (6.53%) was half that reported by the total dietary fiber kit (12.85%). The crude fiber content agreed with previously reported values of 6.13 and 7.02% for *A*. *mexicana* (6, 34). *Atta mexicana* has a fat content comparable to its protein content; for this reason, prior to the fiber determination, a defatting step was required to avoid a coating effect from the fat on the insect matrix, which might also hinder the enzymatic activity. TDF includes soluble and insoluble fiber, and crude fiber is mainly related to the insoluble fraction (35). In the case of insects, the main contribution to the measured values from both techniques is expected to be associated with chitin, which is found in their exoskeletons, and which, upon consumption, functions as a dietary fiber with benefits for gut health after fermentation, cholesterol binding, and immunomodulatory responses (34, 35). The utility of using and reporting enzymatic methods, such as TDF, rather than acid detergent fiber, which does not account for the total insoluble dietary fiber content, has been discussed previously (36), but as shown in Table 1, previous research has only used the crude fiber method. Even though the misconception that chicatana ants directly feed on leaves has led to the characterization of the fiber fraction as acid detergent fiber, hemicellulose, cellulose, and lignin (37). Cellulose, lignin, and hemicellulose are expected in the fiber fraction in small amounts since the acid detergent fraction of insects is primarily made of chitin from the exoskeletons, with a small contribution from retained pigments and proteins (38). Similar to other leaf-cutting ants, *A*. *mexicana* ants collect fresh leaves and transport them to their nests, where they chew them to produce a substrate (pulp) for *Leucoagaricus gongylophorus*, its main feed source, whose enzymes also make leaf nutrients more assimilable and absorbable by the ants (39, 40).

**Table 1:** Proximate content of *Atta mexicana* flour and comparison with other reported values.

Protein content (%)	Fat content (%)	Ash content (%)	Carbohydrates^*^(%)	Crude fiber (%)	Dietary fiber (%)	Processing	Origin	References
38.16 ± 1.40	36.54 ± 1.46	1.91 ± 0.11	10.29 ± 0.27	6.53 ± 0.37	12.85 ± 0.77	Collected, freeze- dried	Huatusco, Veracruz, (2023)	This work
25.81 ± 1.04	51.61 ± 2.11	1.61 ± 0.23	9.69 ± 0.89	11.29 ± 1.18	—	Collected, frozen	Veracruz	(4)
46.00 ± 1.00	39.00 ± 1.00	4.00 ± 1.3	—	11.00 ± 1.4	—	Collected, dry	Puerto Escondido, Oaxaca (1994)	(5)
31.88	34.65	7.58	19.76	6.13	—	Collected, refrigerated	Oaxaca, Oaxaca (2017)	(6)
33.74 ± 1.29	32.00 ± 1.91	4.33 ± 0.54	12.97 ± 0.00	-	—	Dehydrated cooked	Tlaxcala, Tlaxcala (2020)	(8)
66.00	24.02	3.00	4.92	2.06	—	Collected	Mexico	(82)
43.17	38.55	3.75	—	14.12	—	Collected	Chalcatzingo, Morelos (2015)	(37)
43.73	34.15	4.23	9.97	7.92	—	Collected, cooked	Sierra de Huehuetla, Hidalgo	(34)

Data from this work are reported as the mean ± standard deviation (SD) of at least three independent measurements. d.b., dry basis; ^*^Determined by difference (100-moistute-protein-fat-ashes-crude fiber).

In addition to its nutritional composition, standard microbiological analyses were performed as routinely required for different food matrices. Based on the plate count results, the freeze-dried ant sample contained fewer than 10 CFU/g of mesophilic microorganisms (nutrient agar), total coliforms (EMB agar), molds, and yeasts (PDA agar). Therefore, the sample was safe for consumption. Supplementary Figure S11 shows an example of the observed result. This could be related to the specific processing conditions in this study, comprising collection, freezing, washing, and freeze-drying, which reduce the risks associated with regular handling in traditional processing. However, different processing conditions should be studied to eliminate any microbiological hazard.

### 3.2 *In vitro* protein digestibility and chitin content of A. mexicana flour

Table 2 presents the quantified *in vitro* protein digestibility (IVPD) and relative protein digestibility (RPD) of ant flour. The protein concentrate exhibited a higher IVPD and RPD than the ant flour. The alkaline extraction with isoelectric precipitation of Chicatana ant protein yielded a protein concentrate powder equivalent to 43.84% of the original treated ant flour, with a protein content of 42.20%. This extraction method is a widely used conventional alternative in insects due to its simplicity and cost-effectiveness (41, 42). A study of protein concentrates from *A*. *mexicana* in Veracruz found 56.18% protein with antioxidant capacity, as shown by both the concentrate and ant abdomen (4). Moreover, a protein digestibility of 87.6% has been reported for *A*. *mexicana* using the peptidic digestibility method with pepsin (5), which is closer to the value obtained for the protein concentrate in this study (84.67%). An IVPD of 53.95% was also obtained for *Atta laevigata* from Paracambi, Brazil, using pepsin (43). These differences are expected because of variations in the methods and enzymes used. *In vitro* protein digestibility estimates the biological value of the analyzed protein and the availability of its amino acids. Values above 80%, as obtained for the protein from *Atta mexicana*, indicate highly digestible protein, comparable to cooked beef (44). The protein concentrate is expected to show higher values because it lacks interfering molecules (antinutrients or macromolecules). Furthermore, animal-based proteins have higher values than alternative plant proteins (45). The RPD of the ant flour (43.79), compared to casein as a reference protein, was within the range of animal-based proteins (46), while the RPD from the ant protein concentrate increased by 33% compared to the flour value. Proteomic analysis of ant flour identified 2,247 proteins from the abdomen, including peptides with potential antimicrobial and neurological activities. Protein-related bioactivity is mainly linked to free amino acids and peptides, which are breakdown products from digestion (7). *A. mexicana* is also a source of essential amino acids, including histidine, methionine, tryptophan, phenylalanine, threonine, arginine, lysine, isoleucine, valine, and leucine (8).

**Table 2:** *In vitro* protein digestibility (IVPD), relative protein digestibility (RPD), and chitin content of *A. mexicana* whole flour and protein extract.

Sample	IVPD (%)	RPD (%)	Chitin (%)
Whole ant flour	79.06 ± 0.26 ^b^	43.79 ± 0.40 ^b^	16.53 ± 2.53
Protein extract	84.67 ± 0.26 ^a^	66.67 ± 2.40 ^a^	ND

Data are reported as the mean ± standard deviation (SD) of at least three independent measurements.

Different letters (“a” or “b”) indicate significant differences (*p* < 0.05) by Student's *t*-test.

Table 2 also shows the chitin content (16.53%). Chitin contents of 4.18 and 16.05% have been reported for the abdomen of Chicatana ants from Veracruz (4). Chitin is regarded as a dietary fiber; however, because it interferes with protein digestion, it is often classified as an antinutrient (7). Occasionally, fiber content is used as an indirect method to estimate chitin content (47). Researchers should consider potential interferences and fully characterize the dietary fiber fractions in this species. In insects, chitin is part of the exoskeleton, respiratory system, and digestive system. Based on other sources, chitin may have pre-biotic effects, but more research is needed, as, unlike fungal chitin-glucan, this polysaccharide is commonly bound to insect proteins (48).

### 3.3 Relative fatty acid composition of ant flour

Figure 1 presents the fatty acid composition, relative abundance, and enrichment metabolomics of Chicatana ant flour. Additional information on relative areas, numerical abundance, and chromatograms is given in Supplementary Tables S2, S3, and Supplementary Figure S12. The data suggest that these lipids positively contribute to cardiovascular health, anti-inflammatory effects, and cellular health, with particular emphasis on the essential omega-6 fatty acid and oleic acid. Because two lipid extraction methods were used, we compared the lipid profiles obtained by the Soxhlet and Goldfish methods.

**Figure 1:**
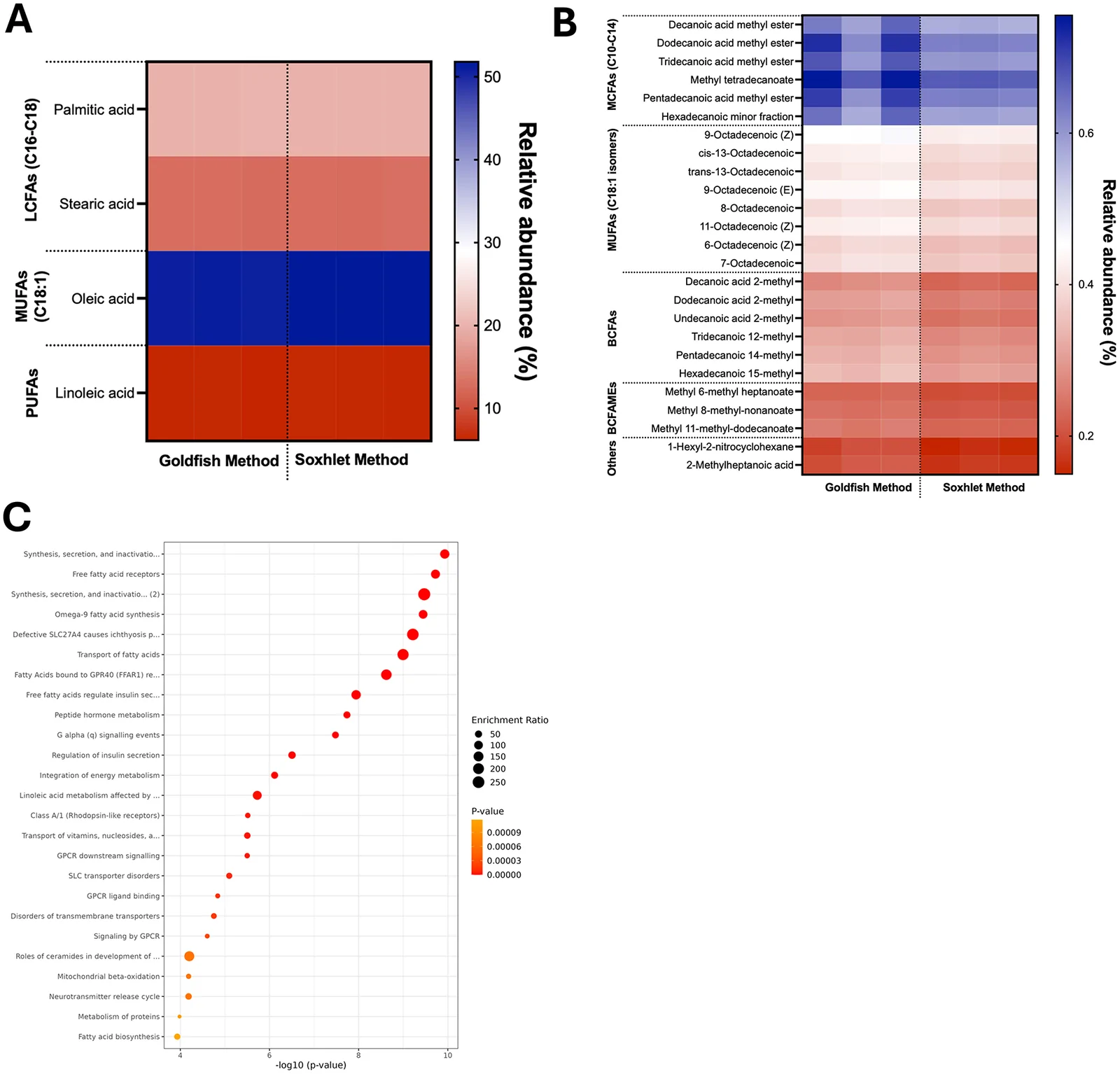
Fatty acid composition of chicatana ant's oil extracted by the Goldfish and Soxhlet methods. **(A)** Relative abundance of the most abundant fatty acids; **(B)** Relative abundance of the least abundant fatty acids; **(C)** Bioinformatics Enrichment metabolite analysis of the obtained lipid profile. Results in **(A)** are reported as mean ± S.D. of at least three extractions in triplicate. Different letters express significant differences (*p*<*0.05*) by Tukey-Kramer's test. BCFAs, branched-chain fatty acids; BCFAMEs, branched-chain fatty acid methyl esters; LCFAs, long-chain fatty acids; MCFAs, medium-chain fatty acids; MUFAs, medium-chain fatty acids; PUFAs, polyunsaturated fatty acids.

*A. mexicana* flour showed a major abundance of four fatty acids: two long-chain fatty acids (palmitic and stearic acids), one monounsaturated fatty acid (MUFAs; oleic acid), and one polyunsaturated fatty acid (PUFAs; linoleic acid; Figure 1A). Among these, oleic acid showed the highest relative abundance (Goldfish: 56.70 ± 0.03%; Soxhlet: 57.02 ± 0.07%), being different between the extraction methods (*p* = *0*.*002*), followed by palmitic acid (Goldfish: 22.26 ± 0.13%; Soxhlet: 21.94 ± 0.12%), which also showed differences (*p* = *0*.*035*). Linoleic acid yielded the lowest relative abundance with no significant differences (Goldfish: 6.95 ± 0.08%; Soxhlet: 6.98 ± 0.08%, *p* = *0*.*64*; Figure 1A).

Regarding the less abundant fatty acids (Goldfish: 10.36 ± 0.35%; Soxhlet: 9.30 ± 0.10%), a collection of medium-chain fatty acids (6 fatty acids), MUFAs (8 fatty acids), branched-chain fatty acids (6 fatty acids), branched-chain fatty acid methyl esters (6 fatty acids), and other compounds (2 compounds) were identified using bioinformatics analysis, considering the GC/MS results and the estimation of their abundances (Figure 1B). Differences were observed by extraction method. Although 11-octadecenoic (0.19 ± 0.01%) and tridecanoic 12-methyl acids (0.73 ± 0.04%) were the least and most abundant fatty acids, respectively, in Goldfish-extracted fat, 1-hexyl-2-nitrocyclohexane (0.15 ± 0.01%) and methyl tetradecanoate (0.67 ± 0.001%) accounted for the least and most abundant, respectively, in Soxhlet-extracted fat.

As shown in Figure 1C, compound enrichment analysis identified associations between the fatty acids and potential functional annotations but not with gene expression or metabolic behavior. Strong associations included fatty acid-mediated signaling, including free fatty acid G protein-coupled receptors and omega-3 and omega-6 fatty acid metabolism.

The abundance of oleic acid suggests its beneficial cardiovascular effects, given its reported metabolic properties (49). This may contribute to lipid quality in insect-based food products. Palmitic acid is another key lipid, with essential roles in maintaining cellular integrity, but its presence suggests a need for dietary balance, given its classification as a saturated fat (50). Stearic acid is a non-harmful saturated fat with anti-inflammatory properties, making it a favorable option compared to other saturated fats in human nutrition (51). Linoleic acid, an essential PUFA, is critical in maintaining skin properties, reducing inflammation, and supporting brain and cardiovascular functions (49). The presence of omega-6 in this lipid profile suggests potential health benefits and highlights the balanced omega-6-to-omega-3 ratio.

Studies on chicatana ants from Tlaxcala have indicated the presence of saturated acids, such as palmitic, myristic, and stearic acids, with oleic and palmitoleic acids as major MUFAs, and linolenic and linoleic acids as the main PUFAs (8). The presence of palmitoleic, linoleic, linolenic, oleic, palmitic, myristic, stearic, and lauric acids has been reported in other edible ant species, including *Carebara vidua, Oecophylla smaragdina, Liometopum apiculatum*, and *Polyrhachis vicina* (52). Other ant species, including *A. laevigata*, also contain oleic, linoleic, and palmitic acids as the main fatty acids, in addition to lauric, myristic, stearic, and arachidic acids in smaller amounts (43). Insect lipid profiles are linked to their ectothermic lifestyle through regulation of enzymatic activities and thermal homeostasis (8). As such, most biological mechanisms in the enrichment analysis indicate that these lipids are strongly associated with lipid signaling and regulatory functions at several biological levels, from receptors in the small intestine and targeted organs to metabolic and endocrine regulation that improves consumers' anti-inflammatory and antioxidant profiles (53). Additional pre-clinical experiments testing these properties could elucidate specific biological mechanisms. Assays on raw and roasted chicatana ants from Veracruz have indicated that the fatty acid profile remains unaffected by the roasting process used for consumption (9). The richness and diversity of fatty acids in the lipid fraction of chicatana ants present a promising avenue for exploring their bioactivity upon digestion and their functionality in product development.

The USDA (54) recommends a daily oleic acid intake of 15 g to reduce the risk of developing coronary heart disease. Hence, a 10–15 g portion of chicatana ants would not be sufficient to meet this recommended value. The nutrient profiles of insect species have been linked to their developmental stage, sex, geographic distribution, and diet (52). As people only consume winged females, their high fat content corresponds with the biological trait of leaf-cutting ants. Females accumulate fat as reserve energy prior to the formation of a new colony, which is a food deprivation period for them (41). Many reports on other ant species correlate the fatty acid profile with the food ants consume, as observed in other insects (55). Regional variability may reflect differences in food sources and environmental mineral content across regions (56). Nevertheless, gaps remain in our understanding of chicatana ants' nutritional content, abiotic factors, and geographic origins that should be addressed.

### 3.4 Microelement and heavy metal contents

The results of ICP-MS of microelements are shown in Table 3. The major microelement in the collected ants was Ca (a macromineral), which aligns with the potential use of insects as a Ca source in complementary diets. Reports on Chinese black ants (*Polyrhachis vicina* Roger) indicate a wide range in Ca content (500–1,100 mg/kg) depending on the collection region (57). Zn, Fe, and Cu are also trace elements found in chicatana ants. Similar values to those determined for chicatana ants in this study were identified for microminerals, such as Mn (17.65 mg/kg), Fe (67.16 mg/kg), Cu (12.94 mg/kg), and Zn (286.1 mg/kg), in *A. laevigata* (43). From a nutritional point of view, Zn is essential for enzymatic and cellular activities, Fe is needed to prevent anemia, and Cu is important for overall growth and Fe absorption. In weaver ants (*Oecophylla smaragdina*), 194.1 mg/kg Fe, 89.48 mg/kg Zn, and 0.43 mg/kg Cu have been reported (58). *Liometopum apiculatum* has shown values of 50 mg/kg Fe, 100 mg/kg Zn, and 260 mg/kg Ca (8). Chicatana ants had a Mn content of 7.60 mg/kg; this trace element supports bone formation, reproduction, and brain function (59). Mn is necessary for some insect functions; overexposure may occur through agricultural spray applications containing MnSO_4_, which alter insect behavior and ecological interactions (60). Selenium in chicatana ants reached 0.17 mg/kg. In its organic form, this metalloid is a dietary element necessary for protection against diseases in animals, including insects. However, human activities can cause overexposure and contamination by increasing selenium levels in ants' diets. Such events affect ant behavior, reproduction, and mortality, as arthropods bioaccumulate toxic compounds, including selenium (61). Additionally, it is necessary to determine whether selenium is present in an organic or inorganic form in edible insects. Based on the FDA guidelines (62), daily dietary intake values for Ca (1,300 mg), Zn (11 mg), Fe (18 mg), Cu (0.9 mg), Mg (420 mg), and Mn (2.3 mg) in adults, a taco or sauce made with 10–15 g of chicatana ants would be equivalent to much lower values than the recommended intake. Therefore, other foods should complement the diet to meet the recommended dietary values.

**Table 3:** Microelemental analysis in freeze-dried *A. mexicana* (mg/kg, d.b.).

Mineral classification	Mineral	Content (mg/kg, d.b.)
Macrominerals	Ca	532.14
Microminerals (trace elements)	Mn	7.60
	Fe	93.92
	Cu	20.43
	Zn	161.09
Heavy metals	As	< 0.53
	Se	0.17
	Cd	< 0.53
	Hg	< 0.03
	Pb	0.10

The symbol “ < ” means that the value was below the analytical quantification limit.

Regarding heavy metals, the detected values for arsenic (< 0.53 mg/kg), cadmium (< 0.53 mg/kg), and mercury (< 0.03 mg/g) were below the method's LOQ. For crustaceans, the European Union (EU) maximum permitted levels for cadmium, mercury, and lead are 0.50 mg/kg (63, 64). The EU maximum permitted levels for lead and cadmium in approved dried and frozen insects are 0.05 and 0.06 mg/kg, respectively, for house crickets (*Acheta domesticus*) (65), 0.07 and 0.05 mg/kg for migratory locusts (*Locusta migratoria*) (65), and 0.1 and 0.05 for lesser mealworm larvae (*Alphitobius diaperinus*). The EU (66) set maximum levels for lead (0.02 mg/kg), cadmium (0.1 mg/kg), mercury (0.005 mg/kg), and arsenic (0.05 mg/kg) in yellow mealworm larvae (*Tenebrio molitor*) (63). The European regulation only contemplates approved edible insects, whereas Mexico's regulatory framework does not include edible insects in its food and food processing regulations. Nevertheless, the lead content in the chicatana ant sample (0.1 mg/kg) exceeded the maximum permissible levels for approved edible insects in the EU. Exploring the main causes and mechanisms of heavy metal exposure is important when addressing food safety in edible insects. Ants are exposed to contaminated environments, in which workers accumulate metals; however, accumulation rates differ among species, life cycles, and castes (67). Heavy metals contaminate not only ants but also their nests because of exposure to different anthropogenic activities (industry, energy generation, transportation, etc.). This relates to the geographical proximity of these activities and whether the regions are developed or undeveloped. In fact, ants are bioindicators of different soil contaminants (68). As noted, wild harvesting of chicatana ants carries a high risk of contaminant exposure, as this activity occurs in both rural and urban areas where heavy metals can accumulate. A critical aspect of this practice is infant consumption of chicatana ants, whose maximum permitted heavy metal levels are even lower because of the physiological risks at such ages. After consuming potentially contaminated insects, lead can accumulate in blood and organs through the soil–plant–insect–animal chain (69). Even at low concentrations, lead can have adverse effects and damage the neurological, reproductive, endocrine, gastrointestinal, respiratory, and cardiovascular systems (70). A critical aspect is that, in environmental research, ants are used to forecast the presence of heavy metals in soil, as heavy metals transfer from soil into ants' bodies, where they bioaccumulate (71). Thus, the bioaccumulation of contaminants, such as heavy metals, in the bodies of chicatana ants and other edible insects is an urgent issue to be considered as part of food safety controls before consumption.

### 3.5 Free phenolic compounds and antioxidant capacity in ant flour

Whole ant flour displayed the highest total phenolics and DPPH scavenging levels, whereas the lipidic fraction showed the opposite (Table 4). We quantified these compounds based on their reported antioxidant and anti-inflammatory effects. Their contents in insect flour remain underexplored and warrant additional research, as some reports have found significant biological potential in these compounds in insects (72). Depending on the extraction method and treatment, chicatana ants have 692–2,279 mg GAE/100 g of extract. Such values were quantified in concentrated extracts prepared by stirring (120 h min) 5 g of flour in 50% ethanol (50 ml), followed by treatments at different pH values (4–8) and temperature combinations (64–95 °C, 15 s−30 min) (73). TPC values in this study were lower than those reported for other well-known and widely consumed insects in Mexico, such as maguey worms (*Aegiale hesperiaris*; 1,346–2,717 mg GAE/100 g extract) and adult grasshoppers (*Splenarium purpurascens*; 575–1,308 mg GAE/100 g extract) (73), as well as other EU-approved species, such as black soldier fly (*Hermetia illucens*) (517 mg GAE/100 g dry matter) and *Tenebrio molitor* (970 mg GAE/100 g dry matter) (74). Regarding DPPH and ABTS, inhibition percentages were similar to those reported in other studies, ranging from 35.97 to 96% for DPPH values (73), yet very wide ranges have been reported for black ant (*Polyrhachis vicina*; 35.21–91.76 %) (75), with the joint contribution of a collection of antioxidant compounds, such as cinnamic acid, quercetin dihydrate, rutin, hesperetin, ferulic acid, and *o-*coumaric acid, contributing to these values in *A*. *mexicana*. For instance, correlations between total phenolic compounds or total flavonoids and the antioxidant capacity have been linked to ABTS and DPPH scavenging values in *Acheta domesticus* and *Tenebrio molitor*, but the potential contribution of amino acids and bioactive peptides could also be considered (76).

**Table 4:** Total phenolic content (TPC) and antioxidant capacity by DPPH scavenging.

Sample	TPC (mg GAE/100 g sample)	DPPH scavenging		ABTS scavenging	
		(mmol TE/100 g sample)	ARA (%)	(mmol TE/100 g sample)	ARA (%)
Whole ant flour	406.36 ± 15.13^a^	38.93 ± 0.17^a^	67.33 ± 1.57^a^	25.30 ± 0.25^a^	72.33 ± 1.20^a^
Defatted ant flour	354.84 ± 4.18^b^	30.54 ± 0.62^b^	61.06 ± 0.79^b^	19.85 ± 0.40^b^	66.18 ± 0.80^b^
Ant fat	32.55 ± 2.34^c^	25.61 ± 1.23^c^	50.35 ± 0.22^c^	16.65 ± 0.75^c^	55.09 ± 0.30^c^

Results are reported as mean ± standard deviation (SD) of three independent measurements. Different letters (“a”, “b” or “c”) indicate significant differences (*p* < 0.05) by Tukey-Kramer's test. ABTS, 2,2-azino-bis (3-ethylbenzothiazoline-6-sulfonic acid); ARA, antiradical activity; GAE, gallic acid equivalents; DPPH, 2,2-diphenyl-1-picrylhydrazyl; TE, trolox equivalents. The defatted flour was obtained by Soxhlet extraction.

### 3.6 Impact of ant flour on the metabolic activity of Caco-2 cells

None of the samples at the tested concentrations were cytotoxic to Caco-2 cells, as indicated by the metabolic activity (>80%; Figure 2A). Defatted samples showed increased activity (*p*<*0*.*05*) at the highest concentrations (+61.40% and +46.85% at 500 and 750 μg/ml, respectively) compared to whole flour. Overall, the cells exposed to the ant's fat exhibited the lowest metabolic activities, but no differences (*p* > *0*.*05*) were observed in the 62.5–750 μg/ml range. Compared with pro-oxidant conditions (Figure 2B), cells exposed to defatted flour showed higher metabolic activity (Supplementary Table S4), suggesting greater cytoprotection. For the other samples (whole flour and fat), we observed no differences under pro-oxidant conditions. These results are consistent with the representative images in Figure 2C, which show no evident signs of cell death at any tested concentration, supporting the safety of these concentrations in Caco-2 cells. Because the fat did not exhibit cytotoxic effects in Caco-2 cells, subsequent tests used the flour rather than the fat.

**Figure 2:**
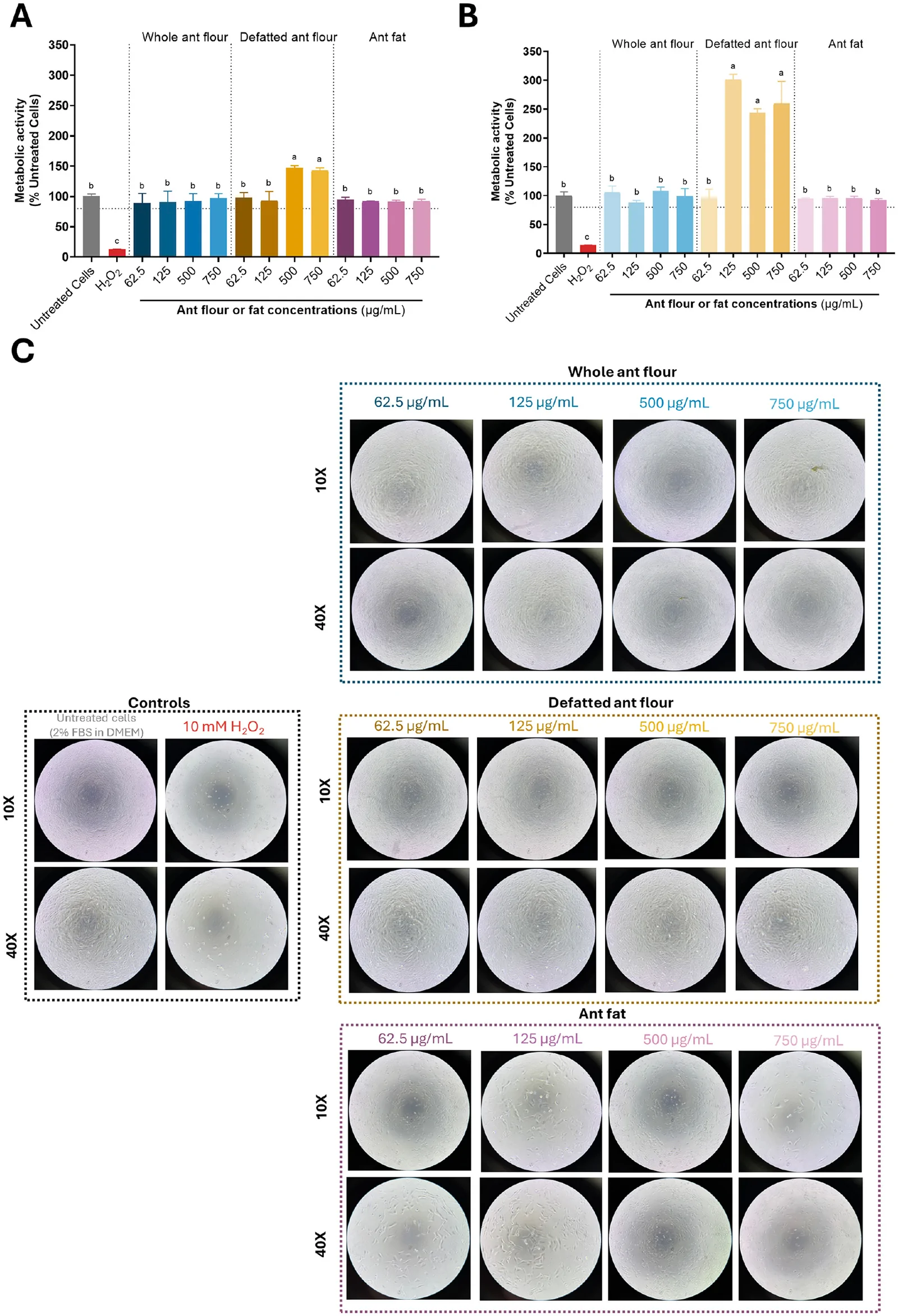
Effect of whole and defatted ant flours, and ant fat on human Caco-2 colorectal cancer cells. **(A)** Metabolic activity under basal conditions; **(B)** Metabolic activity under pro-oxidant conditions; **(C)** Representative pictures of the cells after 24 h exposure to whole and defatted flours. Results in **(A, B)** are reported as mean ± S.D. of two independent experiments in triplicate. Different letters express significant differences (*p*<*0.05*) by the Kruskal–Wallis test, followed by Dunn's test. Untreated cells (negative control) corresponded to 2 % FBS-DMEM-only treated cells, while H_2_O_2_ (positive control) corresponded to 50 mm H_2_O_2_-treated cells.

Previous reports have not considered the effects of *A*. *mexicana* flour on Caco-2 cells, and most reports on other insects have focused on alcoholic extracts or protein hydrolysates in selected cancer cell lines. Recently, assessment of *Tenebrio molitor* aqueous extracts showed elevated IC_50_ values on Caco-2 and hepatic cancer cells (HepG2; 11.44 and 20 mg/ml, respectively), and the prior death mechanism was linked to programmed cell death (apoptosis) mediated by caspase-8 and caspase-9, which induced DNA damage by caspase-3 activation (77). *Tenebrio molitor* oil, which is rich in oleic (827.60 mg/g) and linoleic (505.59 mg/g) acids, among others, inhibited Caco-2 (~90%), HepG2 (~50%), and human cervical cancer (HeLa) cell populations (~15%) at concentrations of 0.1 to 1%, activated pro-apoptotic mechanisms, and caused DNA damage (78).

### 3.7 Anti-inflammatory effect of ant flours in THP-1 cells

An examination of the cytotoxicity of the samples in non-cancerous cells using differentiated macrophages to further assess their anti-inflammatory effects (Figure 3). At the tested concentrations (62.5–750 μg/ml of whole and defatted flour), no cytotoxic effects were observed, but the metabolic activity increased (*p*<*0*.*05*) at the highest concentrations (500 and 750 μg/ml), with no differences compared to 62.5 μg/ml (Figure 3A). Regarding anti-inflammatory activity, as indicated by reduced nitrite production (Figure 3B), all samples induced cells to produce less nitrite than the positive LPS control. However, 125–750 μg/m displayed the lowest production. The IC_50_ of whole ant flour was 39.83% more effective in inhibiting nitrite production (Figure 3C). The IC_50_ of the whole flour contained to 0.021 ± 0.003 mmol/g palmitic acid, 0.012 ± 0.002 mmol/g stearic acid, 0.049 ± 0.008 mmol/g oleic acid, 0.006 ± 0.001 mmol/g linoleic acid, 6.11 ± 0.010 g/ml chitin, and 0.014 ± 0.0005 mg GAE/g sample.

**Figure 3:**
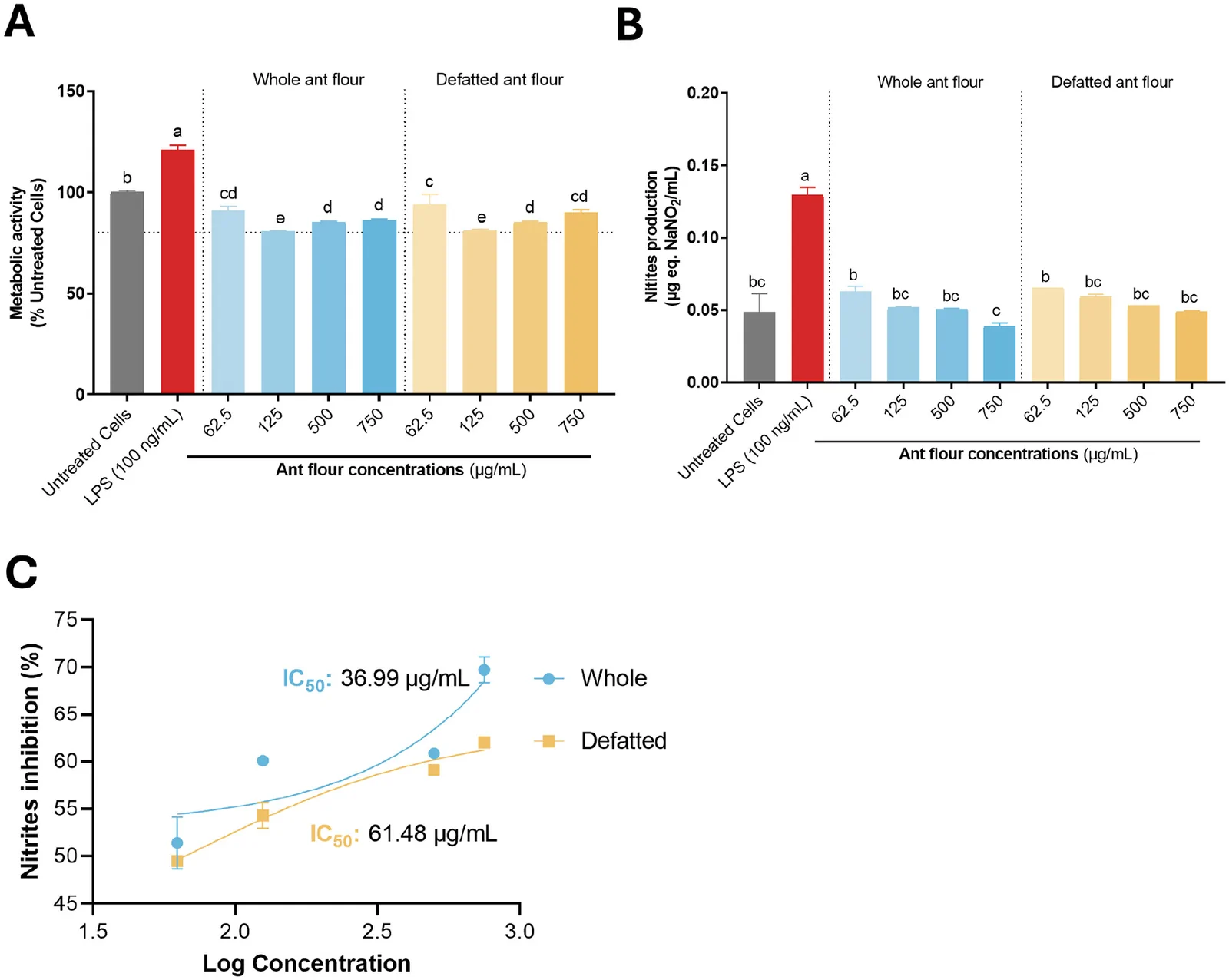
Effect of whole and defatted ant flours on human THP-1 polarized macrophages. **(A)** Metabolic activity under basal conditions; **(B)** Nitrite production quantified by the Griess reaction; **(C)** Calculation of the half-inhibitory concentration (IC_50_) of nitrite production. Results in **(A, B)** are reported as mean ± S.D. of two independent experiments in triplicate. Different letters express significant differences (*p*<*0.05*) by the Kruskal–Wallis test, followed by Dunn's test. Untreated cells (negative control) corresponded to 2 % FBS-RPMI-only treated cells, while LPS (positive control) corresponded to 1 μg/ml LPS-treated cells. LPS: lipopolysaccharides from *E. coli* O111:B4.

Although there are no previous reports on the *in vitro* anti-inflammatory activity of *A*. *mexicana* flour, several bioactive compounds in insects have provided evidence of their anti-inflammatory mechanisms, including phenolic compounds, fatty acids, and bioactive peptides. For instance, an aqueous extract of a beetle species (*Ulomoides dermestoides*), which is presumably rich in several alkenes and terpenes, significantly reduced volume leakage, total leukocytes, and total polymorphonuclear leukocytes in carrageenan-induced pleurisy in rats (8 and 16 mg/kg body weight) (79). Phenolic compounds found in *Vespa orientalis* larval extracts, primarily containing rosmarinic acid, resveratrol, naringenin, and myricetin, reduced intracellular reactive oxygen species, augmented antioxidant enzyme levels (catalase, superoxide dismutase, and glutathione peroxidase), and reduced the nuclear factor kappa B (NF-κB) and IL-8 expression levels (*p*<*0*.*01*), which have been linked to the canonical and cytokines-mediated inflammation pathways (80). A recent characterization of bioactive compounds from black ants (*P. vicina*) extracts identified up to 60 bioactive compounds, mainly fatty acids (particularly long-chain fatty acids: oleic and arachidonic acids), polyphenolic compounds (14 phenolic acids and flavonoids like salicylic acid, *trans-*cinnamic acid, vanillic acid, isoferulic acid, gallic acid; 3,4-dihydroxybenzoic acid, caffeic acid, and naringenin), organic acids (citric acid, ascorbic acid, succinic acid, isocitric acid, and abscisic acid), and nitrogen compounds (amino acids, vitamins, and alkaloids) (75). Collectively, most of these compounds exhibit strong anti-inflammatory effects in polarized THP-1 monocytes through regulation of the NLRP3 inflammasome, critical protein scaffolds related to the exacerbation of inflammation through joint NF-κB and IL-1β activation; and also induce reductions in the production of nitrites, increase antioxidant activity through superoxide dismutase activation and alleviation of intracellular reactive oxygen species, and decrease the production of pro-inflammatory cytokines (29). Isolated non-peptide nitrogen compounds from Chinese black ants (*Polyrhachis dives*) inhibited tumor necrosis factor alpha (TNF-α) production up to 15.71% in LPS-stimulated RW 264.7 murine macrophages, while also exhibiting inhibition of the Jak3 kinase, suggesting anti-inflammatory activity involving potential TNF and Jak receptors, which could affect the production of additional cytokines, such as IL-2, IL-4, IL-7, IL-9, among others (81).

Based on these results, more research is needed on the specific mechanisms of inflammation in *A. mexicana*, as no reports describe inflammation- and antioxidant-associated mechanisms in this ant species. Additionally, results from the *in vitro* assays, although they provide mechanistic and observational insights into potential health benefits, indicate that research on bioaccessibility and bioavailability is needed to quantify and test the extent to which compounds effectively reach the targeted locations where biological activity is needed, including those that remain in the whole matrix after gastrointestinal digestion.

## 4 Conclusions

In addition to its protein content, *A*. *mexicana* (chicatana ant) exhibits a lipid profile containing beneficial fatty acids, particularly oleic and linoleic acids, as well as polyphenolic compounds, with potential implications for antioxidant and anti-inflammatory effects. The presence of both MUFAs and PUFAs, along with relatively low levels of saturated fats, makes this ant species a promising source of high-quality lipids for functional food applications. However, it is important to consider the geographic location, seasonal variation, and dietary intake of Chicatana ants, as studies have shown that these factors significantly influence the profiles of lipids, micronutrients, and bioactive compounds in other insect species. Variations in the environmental conditions and feeding patterns can alter their fatty acid composition and content, affecting the nutritional quality of insect-based products. Future studies should explore the bioavailability and metabolic effects of the macro- and micronutrients identified in this study, while also accounting for external variables to better understand their potential health effects. Despite the high nutritional value of chicatana ants, the whole nutritional assessment of this and other species should integrate the exploration of food safety concerns, including bioaccumulation of food contaminants, good manufacturing practices, and risk assessment and prevention through the food processing chain, based on the identification of lead concentrations above maximum EU-permitted levels for certain edible insects.

## Data Availability

The raw data supporting the conclusions of this article will be made available by the authors, without undue reservation.
