# Supplementary material for: Beyond the role of the reproductive caste of chicatana ants (Atta mexicana) as an alternative protein source: nutritional, food safety, and bioactivity analysis
Source: Front Nutr. 2026 Sep 30;13:1868210. doi: 10.3389/fnut.2026.1868210 (PMC13661416; doi:10.3389/fnut.2026.1868210)
Supplement: Supplementary file 1 [file Table_1.docx]

***Supplementary Material***

Table S1. Limits of detection (LOD) and limits of quantification (LOQ) for the microelemental analysis by ICP-MS

| **Element** | **LOD (ng/ml)** | **LOQ (ng/ml)** |
| --- | --- | --- |
| 44Ca | 5.0 | 250.0 |
| 55Mn | 0.1 | 0.5 |
| 56Fe | 5.0 | 500.0 |
| 63Cu | 0.1 | 0.5 |
| 66Zn | 0.1 | 5.0 |
| 75As | 0.001 | 0.005 |
| 78Se | 0.1 | 1.0 |
| 111Cd | 0.001 | 0.005 |
| 201Hg | 0.1 | 0.5 |
| 208Pb | 0.1 | 0.5 |

Figure S1. Calibration curve for the analysis of Ca by ICP-MS

Figure S2. Calibration curve for the analysis of Mn by ICP-MS

Figure S3. Calibration curve for the analysis of Fe by ICP-MS

Figure S4. Calibration curve for the analysis of Cu by ICP-MS

Figure S5. Calibration curve for the analysis of Zn by ICP-MS

Figure S6. Calibration curve for the analysis of Se by ICP-MS

Figure S7. Calibration curve for the analysis of Pb by ICP-MS

Figure S8. Calibration curve for the analysis of Hg by ICP-MS

Figure S9. Calibration curve for the analysis of As by ICP-MS

Figure S10. Calibration curve for the analysis of Cd by ICP-MS

Figure S11. Results of the analysis of chicatana flour for yeast and molds in PDA at a 10^-2^ (First column), 10^-4^ (Second column), and 10^-6^ (Third column) dilution (n=3).

Figure S12. Chromatogram of fatty acids with retention times

Table S2. GC/MS identification matches with retention times

| **RT (s)** | **Fatty acid name** | **Match**  **(%)** | **Area**  **(u^2^)** | **Area**  **(%)** |
| --- | --- | --- | --- | --- |
| 12.74 | Undecanoic acid* | 98.6 | 132,058,200 | 8.826 |
| 20.91 | Palmitic acid | 98.8 | 298,543,552 | 19.954 |
| 24.13 | Stearic acid | 99.0 | 201,351,920 | 13.458 |
| 25.15 | Oleic acid | 99.9 | 771,740,864 | 51.580 |
| 26.31 | Linoleic acid | 98.4 | 92,498,000 | 6.182 |

*Undecanoic acid was spiked as internal stsandard

Table S3. Fatty acid relative abundance in chicatana ant’s oil extracted by the Goldfish and Soxhlet methods

| **Fatty acid** | **Goldfish**  **(%)** | **Soxhlet**  **(%)** |
| --- | --- | --- |
| Palmitic acid | 22.26 ± 0.13^a^ | 21.94 ± 0.12^b^ |
| Stearic acid | 14.09± 0.12^a^ | 15.05 ± 0.18^a^ |
| Oleic acid | 56.70 ± 0.03^b^ | 57.02 ± 0.07^a^ |
| Linoleic acid | 6.95 ± 0.08^a^ | 6.98 ± 0.08^a^ |

Different letters indicate significant differences (p ≤ 0.05) between extractions for a specific fatty acid from the same row.

Table S4. Metabolic activity under basal and pro-oxidant conditions for untreated, and H_2_O_2_-, whole ant flour-, defatted ant flour-, and fat-treated Caco-2 cells.

| **Treatments** | | **Metabolic activity (% Untreated cells)** | |
| --- | --- | --- | --- |
|  |  | **Basal conditions** | **Pro-oxidant conditions** |
| Untreated cells | | 100 ± 3.97 ^a^ | 100 ± 6.41 ^a^ |
| H_2_O_2_-treated cells | | 12.76 ± 0.31 ^b^ | 14.11 ± 0.62 ^a^ |
| Whole ant flour (µg/mL) | 62.5 | 89.77 ± 15.31 ^a^ | 105.87 ± 10.73 ^a^ |
|  | 125 | 90.96 ± 17.77 ^a^ | 88.31 ± 3.28 ^a^ |
|  | 500 | 92.30 ± 12.38 ^a^ | 108.34 ± 6.49 ^a^ |
|  | 750 | 97.81 ± 6.79 ^a^ | 98.64 ± 13.46 ^a^ |
| Defatted ant flour (µg/mL) | 62.5 | 98.34 ± 8.16 ^a^ | 96.59 ± 14.35 ^b^ |
|  | 125 | 93.62 ± 14.53 | 301.05 ± 9.14 |
|  | 500 | 147.00 ± 4.20 ^b^ | 243.10 ± 7.45 ^a^ |
|  | 750 | 143.11 ± 4.31 ^b^ | 260.20 ± 37.79 ^a^ |
| Ant fat (µg/mL) | 62.5 | 94.83 ± 4.06 ^a^ | 94.62 ± 1.50 ^a^ |
|  | 125 | 92.03 ± 0.79 ^b^ | 95.98 ± 2.56 ^a^ |
|  | 500 | 91.19 ± 2.64 ^b^ | 95.73 ± 3.15 ^a^ |
|  | 750 | 92.19 ± 3.33 ^a^ | 92.15 ± 2.62 ^a^ |

The results are presented as the mean ± S.D. of two independent experiments in triplicate. Different letters by row indicate significant differences (*p<0.05*) by Wilcoxon’s test. Untreated cells refer to Caco-2 cells with no treatment (2 % FBS-DMEM), whereas H_2_O_2_-treated cells refer to 10 mM H_2_O_2_ dissolved in 2% DMEM. Pro-oxidant conditions were simulated using 10 mM H_2_O_2_.
